# Comparative outcomes of neoadjuvant immunochemotherapy versus neoadjuvant chemoradiotherapy in locally advanced thoracic esophageal squamous cell carcinoma: a target trial emulation

**DOI:** 10.3389/fimmu.2026.1916623

**Published:** 2026-09-03

**Authors:** Zhiyu Li, Qiuxi Zhou, Qin Xie, Chenghao Wang, Lin Peng, Yongtao Han, Xuefeng Leng, Yan Miao, Qi Zhang, Wenwu He

**Affiliations:** 1 School of Pharmacy, Faculty of Medicine, Macau University of Science and Technology, Macao SAR, China; 2 Department of Thoracic Surgery, Sichuan Clinical Research Center for Cancer, Sichuan Cancer Hospital & Institute, Sichuan Cancer Center, University of Electronic Science and Technology of China, Chengdu, China; 3 Department of General Internal Medicine, Sichuan Clinical Research Center for Cancer, Sichuan Cancer Hospital & Institute, Sichuan Cancer Center, University of Electronic Science and Technology of China, Chengdu, China; 4 Department of Thoracic Surgery, Leshan People’s Hospital, Leshan, China

**Keywords:** cancer-specific survival, disease-free survival, esophageal squamous cell carcinoma, neoadjuvant chemoradiotherapy, neoadjuvant immunochemotherapy, overall survival, pathological complete response, recurrence-free survival

## Abstract

**Background:**

Neoadjuvant chemoradiotherapy (NCRT) is standard for locally advanced esophageal squamous cell carcinoma (ESCC), while neoadjuvant immune-chemotherapy (NICT) is a promising alternative. This study compared pathological response and long-term survival between NICT and NCRT followed by surgery using a target trial emulation framework.

**Methods:**

We emulated a target trial in 288 consecutive patients with locally advanced thoracic ESCC who underwent neoadjuvant therapy and esophagectomy between March 2018 and December 2022. Eligible patients were aged 18–80 years and had clinical stage cT1N+M0 or cT2–4aN0–3M0 disease. The primary endpoints were overall survival (OS) and disease-free survival (DFS), with cancer-specific survival (CSS) and recurrence-free survival (RFS) as additional endpoints. Baseline characteristics were balanced after inverse probability of treatment weighting (IPTW). Bootstrap-weighted Cox models were applied to small subgroups for exploratory analyses.

**Results:**

Median follow-up was 49.3 months. Both pre- and post-IPTW, the NCRT group experienced a significantly higher incidence of severe (grade 3-4) myelosuppression compared with NICT (P < 0.001), whereas no significant differences were observed between the two cohorts in surgery-related outcomes. In the unadjusted cohort, patients receiving NCRT achieved superior pathological responses, with higher rates of pathological complete response (pCR) and tumor regression grade (TRG). After IPTW, all pathologic endpoints were well balanced except for TRG 0, which remained marginally different (P = 0.051, SMD = 0.376). Before IPTW, there were no significant differences between NCRT and NICT in OS or DFS (OS: HR = 1.08, 95% CI 0.72-1.63, P = 0.701; DFS: HR = 1.02, 95% CI 0.70-1.48, P = 0.915). Although the KM curves for CSS and RFS showed a separation trend, the differences were not significant (CSS: HR = 0.74, 95% CI 0.44-1.23, P = 0.245; RFS: HR = 0.72, 95% CI 0.46-1.14, P = 0.163). After IPTW weighting, OS and DFS remained comparable between the two groups, whereas NICT demonstrated advantages in RFS (HR = 0.47, 95% CI 0.27-0.82, P = 0.008) and CSS (HR = 0.49, 95% CI 0.26-0.89, P = 0.020). Multivariable analysis identified higher ypN stage as independent predictors of worse OS and DFS. Lower ypN stage and receipt of NICT were associated with better CSS and RFS.

**Conclusions:**

Among patients with locally advanced ESCC receiving neoadjuvant therapy, NCRT was associated with superior pathological response but also a higher risk of severe myelosuppression. After IPTW adjustment, NICT and NCRT showed comparable OS and DFS, while NICT was associated with significantly better RFS and CSS. These findings suggest that NICT may be a feasible alternative to NCRT in this population.

## Introduction

Esophageal cancer is the 11th most commonly diagnosed cancer and the seventh leading cause of cancer death worldwide, represents a global health concern (1). Over 40% of cases occur in China, where esophageal squamous cell carcinoma (ESCC) is the most common histological type (2). At the time of initial evaluation, the majority of patients are already diagnosed with locally advanced disease. According to the guidelines from the National Comprehensive Cancer Network (NCCN), neoadjuvant chemoradiotherapy (NCRT) is recommended as the standard treatment for patients with locally advanced ESCC (3). Although the CROSS trial and the NEOCRTEC-5010 trial reported that NCRT provides greater long-term survival benefits compared to surgery alone, over 30% of patients still experience local recurrence or distant metastasis (4, 5). Additionally, the JCOG9907 trial result revealed that neoadjuvant chemotherapy (NCT) followed by surgery was recommended as a therapeutic option (6). While recent studies have demonstrated improvements in the 5-year survival rates (47.0%-60.5%) (4–10) of patients with locally advanced esophageal cancer through NCT or NCRT, the outcomes are still unsatisfactory.

Immune checkpoint inhibitors (ICIs) have demonstrated effectiveness in advanced esophageal cancer (KEYNOTE-590 trial) (11). The postoperative adjuvant therapy using anti-programmed death 1 (PD-1) antibodies after NCRT has also been shown to significantly prolong progression-free survival (CheckMate-577 trial) (12). It is worth noting that a recent large-scale multicenter clinical study reported superior long-term outcomes with NICT compared to NCRT, including a significantly lower risk of recurrence, although only 2-year OS and DFS were reported (13). In contrast, several smaller studies have suggested that NICT may offer long-term survival outcomes that are merely comparable to those of NCRT (14, 15). As a promising neoadjuvant treatment approach, neoadjuvant immunochemotherapy (NICT) has demonstrated good safety and manageable adverse events, but the long-term survival outcomes remain unclear (16–18). Our aim was to compare NICT versus NCRT followed by surgery for therapeutic response and survival, to situate the findings within the existing literature, and to clearly outline the conclusions and limitations, thereby offering meaningful insights into neoadjuvant treatment strategies for locally advanced ESCC.

## Methods

### Patients and target trial emulation

We emulated a target trial to compare two neoadjuvant treatment strategies for patients with locally advanced ESCC: NICT versus NCRT. Patients with locally advanced ESCC who received NICT or NCRT between March 2018 and December 2022 were included. The study included patients meeting the following criteria: (I) aged between 18 and 80 years; (II) diagnosed with thoracic ESCC staged as cT1N+M0 or cT2-4aN0-3M0; (III) underwent neoadjuvant therapy followed by esophagectomy. Exclusion criteria included: (I) had a history of malignancy; (II) did not complete neoadjuvant treatment or required salvage surgery; (III) lacked clinical data or follow-up information. The study was approved by the Ethics Committee (No. SCCHEC-02-2022-050); The requirement for informed consent was waived on account of anonymous data.

### Treatment protocols

Patients received neoadjuvant immunochemotherapy consisting of an immune checkpoint inhibitor administered intravenously at a fixed dose of 200 mg every 3 weeks, given concurrently with a platinum–taxane doublet. The immune checkpoint inhibitors used in this study were anti-PD-1 agents, specifically camrelizumab, nivolumab, pembrolizumab, toripalimab, tislelizumab, and sintilimab. Platinum agents included cisplatin, carboplatin, or nedaplatin; taxanes included paclitaxel or docetaxel. NCRT group patients followed the concurrent NCRT treatment, with a total radiotherapy dose of 40.0 to 50.4 Gy delivered in 1.8 to 2.14 Gy fractions, 5 fractions per week. The chemotherapy regimen was the same as in the NICT group. All patients received McKeown or Ivor Lewis esophagectomy within 4–8 weeks after completing neoadjuvant therapy. Regularly, the 2-field lymphadenectomy was conducted. Three-field lymphadenectomy was conducted for patients having suspected metastatic lymph nodes within the cervical area.

### Causal contrast and time zero

The time zero was defined as the start date of the first neoadjuvant treatment. The causal contrast of interest was the intention-to-treat effect of initiating NICT versus initiating NCRT among eligible patients. Patients were analyzed according to their initially assigned treatment strategy, regardless of subsequent treatment modifications, provided that they completed the planned neoadjuvant course and proceeded to surgery. This design was chosen to reduce time-related bias and to align the analysis with a target trial framework.

### Pathologic analysis

R0 resection was defined as a radical resection with negative distal, proximal, and circumferential resection margins. Pathologic response was assessed using the AJCC 8th edition TRG system (tumor regression grade of American Joint Committee on Cancer). Pathological complete response (pCR) was defined as the absence of any remaining tumor within the esophagus and the resected lymph nodes.

### Outcomes

Following the surgery, patients were assessed every 3 months for the first 2 years, every 6 months in the next 3 years, and yearly thereafter, with documenting survival status, disease progression, and other treatments received at each follow-up. Routine assessments included: (i) contrast-enhanced CT scans of the chest and abdomen to screen for locoregional and distant recurrence; (ii) supplementary MRI or PET/CT when indicated to further characterize any suspicious lesions; (iii) video gastroscopy or esophagoscopy to inspect the anastomotic site and residual esophageal mucosa; and (iv) laboratory tests—complete blood count, serum biochemistry and tumor markers (e.g., CEA, SCC)—to monitor both treatment-related toxicity and tumor activity. Local recurrence was defined as recurrence in the supraclavicular, mediastinal, and peritoneal regions. Distant metastasis was defined as recurrence elsewhere. All relapses were confirmed by CT scan, MRI scan, PET/CT scan or endoscopic examinations at the corresponding sites. Cytology or histology should be performed if necessary. Overall survival (OS): From start of first treatment to death from any cause; censored at last follow-up. Disease-free survival (DFS): From surgery to first recurrence, death, or last follow-up. Cancer-specific survival (CSS): From start of first treatment to death from the index cancer; censored at last follow-up or death from other causes. Recurrence-free survival (RFS): From surgery to first documented recurrence; deaths without recurrence are censored at death. Local recurrence-free survival (LRFS)/distant metastasis-free survival (DMFS): From surgery to first local recurrence/distant metastasis; censored at last follow-up or death from other causes.

### Statistical analysis

Baseline characteristics were summarized using descriptive statistics. Categorical variables were compared using the chi-square test or Fisher’s exact test, and continuous variables were compared using the independent-samples t-test, as appropriate.

To emulate random treatment assignment and reduce confounding, inverse probability of treatment weighting (IPTW) was applied. Covariates were selected *a priori* based on clinical relevance and potential confounding, including age strata, sex, tumor location, clinical T stage, clinical N stage, clinical TNM stage, number of dissected lymph nodes, neoadjuvant cycles, adjuvant therapy, surgical approach, and tumor proportion score (TPS), among others. Stabilized weights were used to improve estimate precision, and extreme weights were trimmed at the 1st and 99th percentiles. The effective sample size (ESS) was calculated as ESS = (Σw)^2/Σ(w^2) to quantify information loss due to weight variability. The R package survey was used for weighted analyses and variance estimation. Covariate balance after weighting was assessed using standardized mean differences (SMDs), with SMD < 0.1 considered acceptable.

Survival curves were estimated using the Kaplan–Meier method, and group comparisons were performed with the log-rank test. Cox proportional hazards models were used to estimate hazard ratios for OS and DFS. Variables with P < 0.05 in univariable analyses were entered into multivariable models. For subgroup analyses with small sample size (n < 20), a bootstrap sensitivity analysis with 2,000 replications was performed. In each bootstrap sample, the original analysis weights were applied and rescaled by their mean; weighted Cox models were then fitted to estimate log hazard ratios. The empirical distribution of successfully fitted estimates was used to derive 95% confidence intervals by the percentile method and two-sided bootstrap P values.

All analyses were performed using R version 4.4.3 with the survival version 3.8–3 and boot version 1.3–31 packages on Windows 10 Pro 64-bit (version 10.0.19044).

## Results

### Patient characteristics

**Figure 1:**
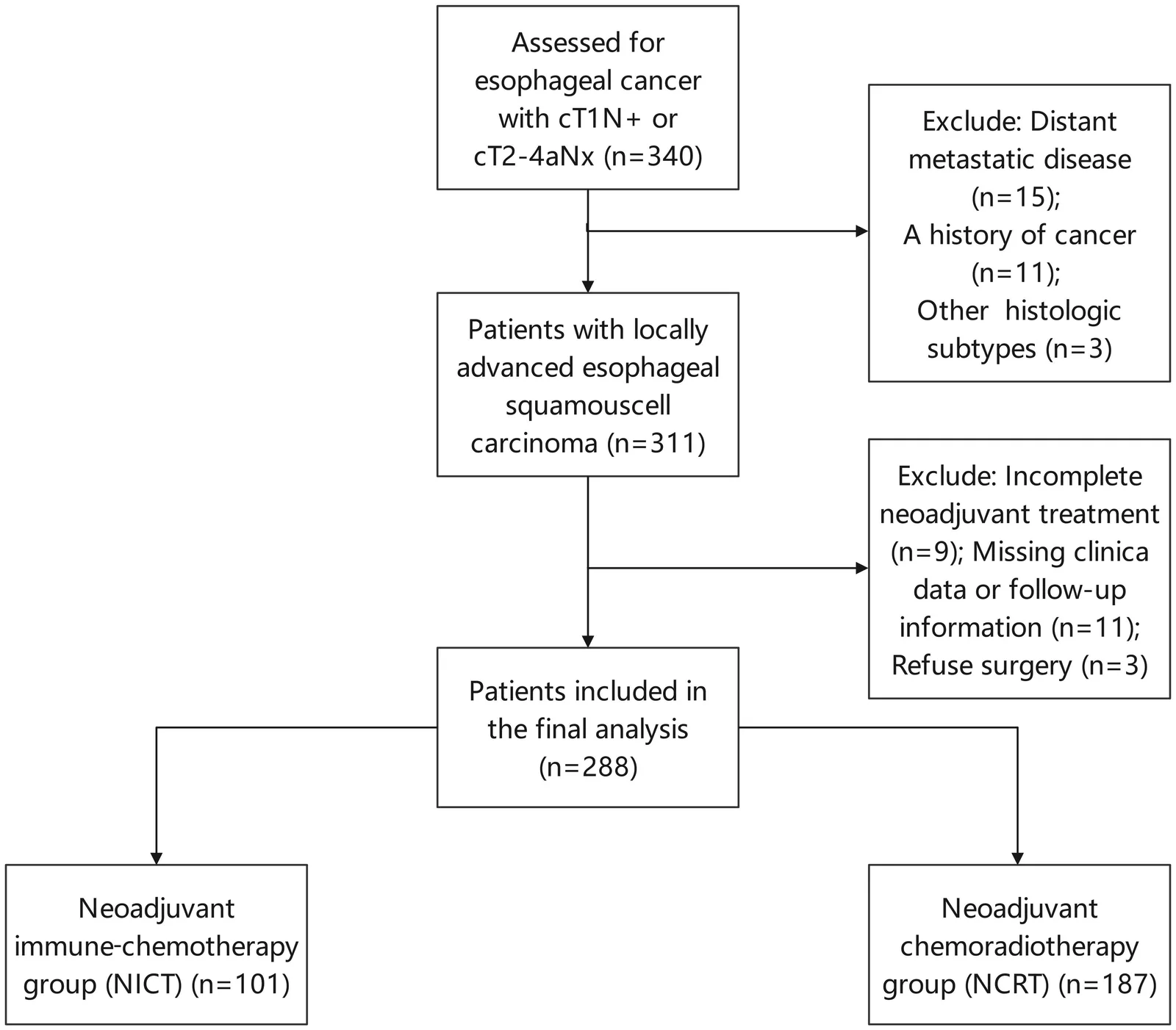
CONSORT diagram. ESCC, esophageal squamous cell carcinoma.

**Figure 2:**
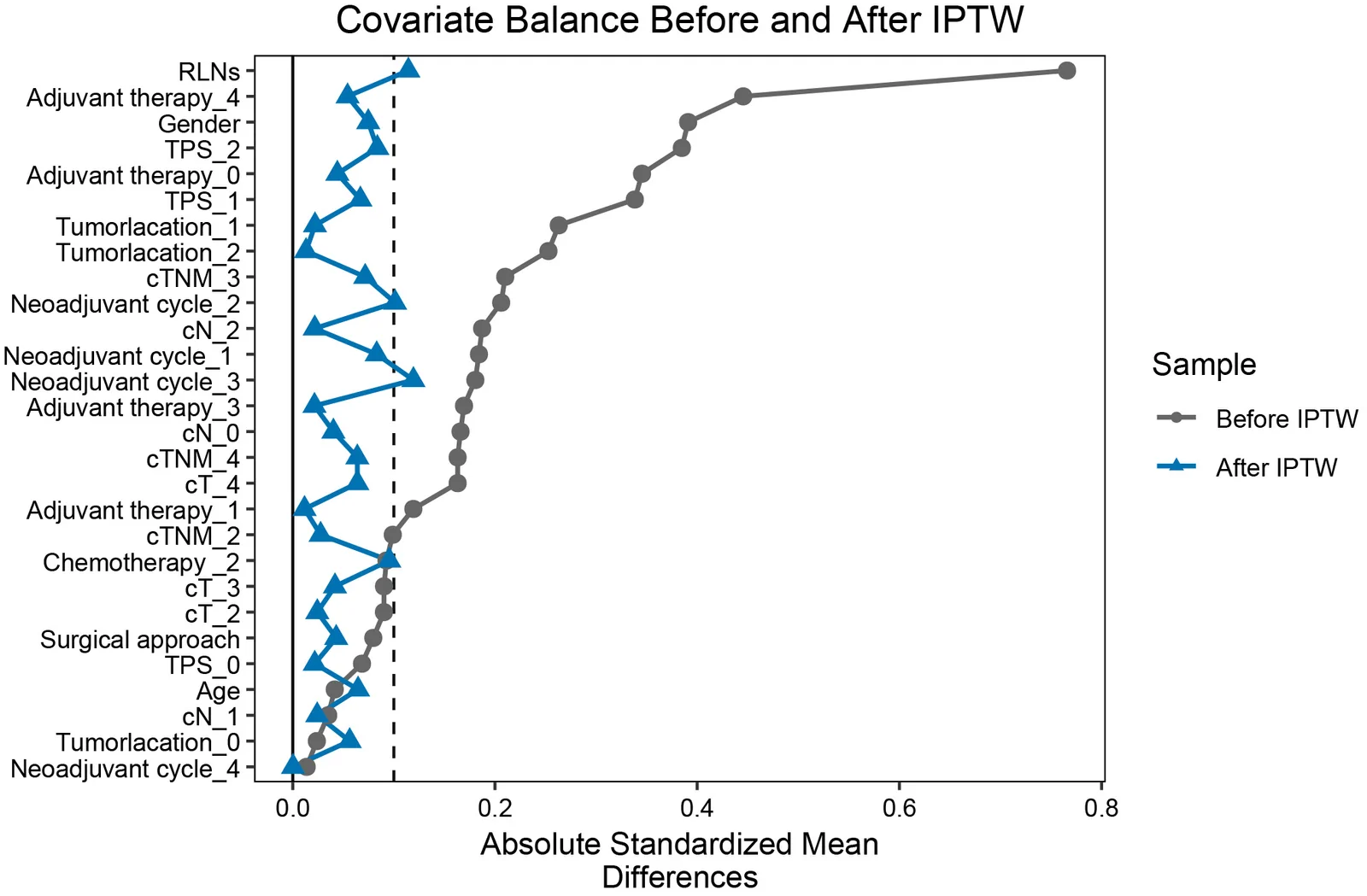
Covariate balance before and after inverse probability of treatment weighting (IPTW).

**Figure 3:**
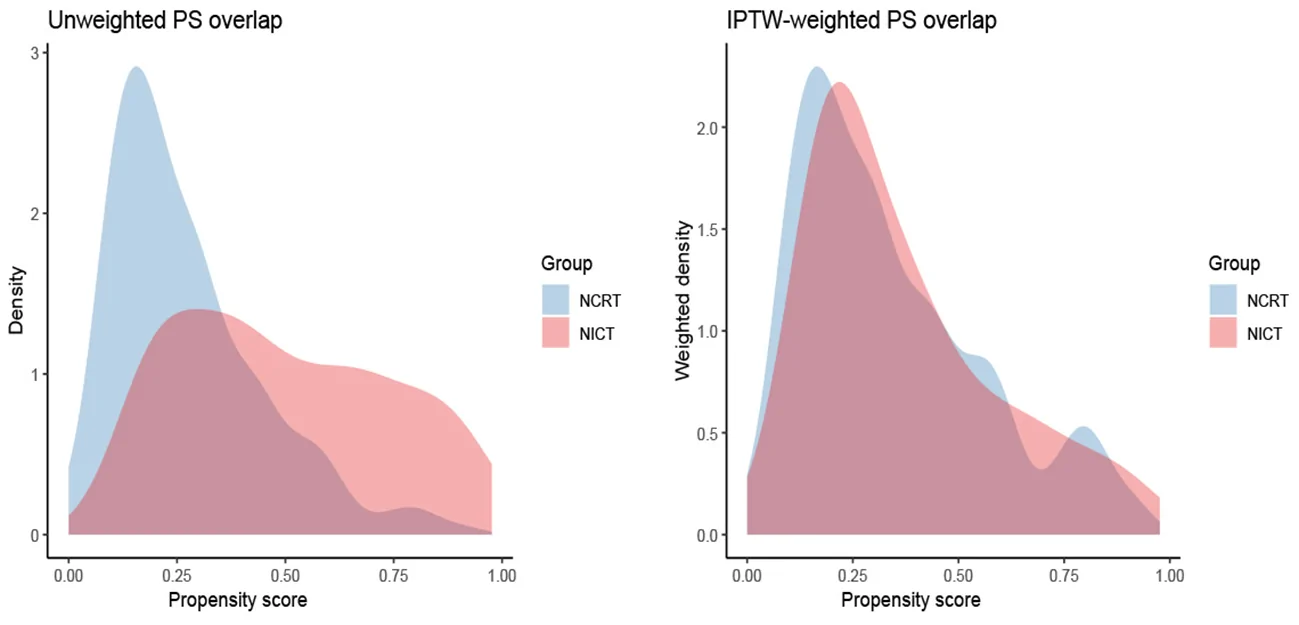
Propensity score distributions before and after inverse probability of treatment weighting (IPTW).

A total of 288 patients from our institution were included in the final analysis (**Figure 1**, CONSORT Diagram), of whom 187 received conventional NCRT and 101 received NICT. Baseline characteristics are summarized in **Table 1**. The NICT group had a greater number of lymph nodes examined (p < 0.001, SMD = 0.766) and a higher TPS (p = 0.005, SMD = 0.413). Among the ICIs used in the NICT group, camrelizumab was the most frequently administered agent (35, 34.7%), followed by toripalimab (31, 30.7%), pembrolizumab (12, 11.9%), tislelizumab (12, 11.9%), sintilimab (9, 8.9%), and nivolumab (2, 2.0%). Most patients received two neoadjuvant cycles (95, 94.1%). The overall weight had a mean of 0.971, SD 0.500, median 0.823, and interquartile range 0.721-1.066; the 1st and 99th percentiles were 0.370 and 3.368, respectively, and the minimum/maximum values were 0.370 and 3.411. The results indicate some tail heaviness in the weights, with extreme values controlled via trimming (the love plot and overlap plots are shown in **Figures 2** and **3**, the log–log survival plot (**Supplementary Figure 6**) and Schoenfeld residual plots (**Supplementary Figure 7**) are provided in the **Supplementary Materials**).

**Table 1:** Patient characteristics.

	Before IPTW				After IPTW			
	NICT	NCRT	p	SMD	NICT	NCRT	p	SMD
Variables	n=101	n=187			n=95.9	n=183.7		
Gender (%)								
Male	53 (52.5)	133 (71.1)	0.002	0.391	59.9 (62.4ff)	121.3 (66.0)	0.596	0.074
Female	48 (47.5)	54 (28.9)			36.0 (37.6)	62.5 (34.0)		
Age (%)								
≤60	48 (47.5)	85 (45.5)	0.832	0.042	46.9 (49.0)	84.0 (45.7)	0.65	0.065
>60	53 (52.5)	102 (54.5)			48.9 (51.0)	99.7 (54.3)		
Tumor location (%)								
Upper	11 (10.9)	19 (10.2)	0.091	0.272	11.8 (12.3)	19.5 (10.6)	0.931	0.054
Middle	43 (42.6)	104 (55.6)			48.6 (50.7)	95.2 (51.8)		
Lower	47 (46.5)	64 (34.2)			35.4 (36.9)	69.0 (37.6)		
cT (%)								
T2	7 (6.9)	9 (4.8)	0.361	0.179	5.4 (5.6)	9.3 (5.1)	0.885	0.066
T3	82 (81.2)	145 (77.5)			77.4 (80.7)	145.2 (79.0)		
T4a	12 (11.9)	33 (17.6)			13.1 (13.7)	29.3 (15.9)		
cN (%)								
N0	33 (32.7)	76 (40.6)	0.193	0.221	34.6 (36.1)	69.9 (38.0)	0.961	0.041
N1	52 (51.5)	93 (49.7)			46.9 (48.9)	87.7 (47.7)		
N2/3	16 (15.8)	18 (9.6)			14.4 (15.0)	26.2 (14.3)		
cTNM (%)								
II	32 (31.7)	68 (36.4)	0.197	0.226	31.2 (32.5)	62.1 (33.8)	0.849	0.08
III	57 (56.4)	86 (46.0)			51.6 (53.9)	92.4 (50.3)		
IVA	12 (11.9)	33 (17.6)			13.1 (13.7)	29.3 (15.9)		
Surgical approach (%)								
McKeown	94 (93.1)	170 (90.9)	0.682	0.08	89.0 (92.8)	168.3 (91.6)	0.748	0.043
Ivor Lewis	7 (6.9)	17 (9.1)			6.9 (7.2)	15.4 (8.4)		
RLNs (mean (SD))	26.49 (12.58)	18.26 (8.52)	<0.001	0.766	21.90 (10.74)	20.67 (9.59)	0.387	0.121
TPS (%)								
< 1%	29 (28.7)	48 (25.7)	0.005	0.413	26.4 (27.5)	48.7 (26.5)	0.834	0.085
≥1%	40 (39.6)	45 (24.1)			31.2 (32.5)	54.1 (29.4)		
Unknown	32 (31.7)	94 (50.3)			38.4 (40.0)	80.9 (44.0)		
Chemotherapy regimen								
TP	100 (99)	183 (97.9)	0.811	0.093	95.1 (99.1)	180.1 (98.0)	0.403	0.101
CF	1 (1)	4 (2.1)			0.8 (0.9)	3.7 (2.0)		
Dose of radiation (mean (SD))	NA	40.04 (2.6)	NA	NA	NA	40.02 (2.3)	NA	NA
ICIs								
Camrelizumab	35 (34.7)	NA	NA	NA	36.7 (38.3)	NA	NA	NA
Nivolumab	2 (2.0)	NA			2.5 (2.6)	NA		
Pembrolizumab	12 (11.9)	NA			8.5 (8.8)	NA		
Toripalimab	31 (30.7)	NA			29.4 (30.6)	NA		
Tislelizumab	12 (11.9)	NA			11.1 (11.6)	NA		
Sintilimab	9 (8.9)	NA			7.7 (8.1)	NA		
Neoadjuvant cycles (%)								
1	3 (3.0)	13 (7.0)	0.29	0.263	3.6 (3.7)	10.2 (5.5)	0.683	0.171
2	95 (94.1)	165 (88.2)			89.5 (93.3)	166.2 (90.5)		
3	0 (0.0)	3 (1.6)			0.0 (0.0)	1.9 (1.1)		
4	3 (3.0)	6 (3.2)			2.8 (2.9)	5.4 (2.9)		
Adjuvant therapy (%)								
None	73 (72.3)	161 (86.1)	<0.001	0.504	77.9 (81.3)	152.6 (83.0)	0.969	0.068
Chemotherapy	3 (3.0)	10 (5.3)			4.1 (4.3)	8.2 (4.5)		
Chemoradiotherapy	12 (11.9)	13 (7.0)			8.2 (8.6)	14.6 (8.0)		
Immunotherapy	13 (12.9)	3 (1.6)			5.6 (5.9)	8.3 (4.5)		

Data are presented as n (%); NICT, Neoadjuvant immuno-chemotherapy; NCRT, neoadjuvant chemoradiotherapy; RLNs, retrieved lymph nodes; TPS, tumor proportion score; IPTW, Inverse probability of treatment weighting; SMD, Standardized mean differences; TP, Taxane + Platinum regimen; CF, Cisplatin + 5-Fluoropyrimidine.

The overall ESS was 227.8. By group, the NCRT group had a total weighted sum of approximately 183.7 and an ESS of 154.7 (efficiency = ESS/weighted sum ≈ 84%), while the NICT group had a total weighted sum of approximately 95.9 and an ESS of 73.6 (efficiency ≈ 77%). Compared with the total weighted sums, the ESS values were slightly lower, indicating some variability in weights; however, the observed efficiencies fall within a common and acceptable range. After IPTW adjustment, clinical characteristics were well balanced between the two groups (**Table 1**).

### Neoadjuvant therapy toxicity and perioperative outcomes

In the NICT group, 3 received only one cycle of neoadjuvant therapy. In the NCRT group, 13 received one cycle of neoadjuvant chemotherapy, and 6 patients did not complete the planned course of radiotherapy. Due to myelosuppression, 1 in the NICT group switched to oral S-1 (tegafur), and 2 patients in the NCRT group also switched to oral S-1. Additionally, because of a drug allergy, 1 in the NCRT changed the regimen from CF to TP. Severe hematologic toxicity was more frequent in the NCRT group than in the NICT group, particularly leukopenia, neutropenia, and lymphocytopenia. In contrast, grade 3–4 thrombocytopenia, dermatitis, and hepatic dysfunction were uncommon and did not differ meaningfully between groups. Other perioperative outcomes, including operative time, blood loss, postoperative pulmonary infection, pleural effusion, chylothorax, recurrent laryngeal nerve injury, anastomotic leak, length of stay, and unplanned readmission, were comparable between groups. There were no 30-day postoperative deaths in either group, and one 90-day death occurred in the NCRT group. No severe immune-related adverse events were observed in the NICT group. Details are provided in **Table 2**.

**Table 2:** Neoadjuvant therapy toxicity and perioperative outcomes.

	Before IPTW				After IPTW			
Variables	NICT	NCRT			NICT	NCRT		
	101	187	p	SMD	95.9	183.7	p	SMD
Leukopenia (level 3-4, %)	2 (2.0)	29 (15.5)	<0.001	1.502	4.2 (4.4)	27.3 (14.8)	<0.001	1.427
Neutropenia (level 3-4, %)	3 (3.0)	26 (13.9)	<0.001	0.863	3.1 (3.3)	25.5 (13.9)	<0.001	0.809
Lymphocytopenia (level 3-4, %)	6 (6.0)	120 (64.2)	<0.001	2.006	5.5 (5.8)	121.4 (66.1)	<0.001	2.067
Thrombocytopenia (level 3-4, %)	0 (0.0)	2 (1.1)	0.244	0.276	0.0 (0.0)	1.8 (1.0)	0.606	0.206
Dermatitis (level 3-4, %)	0 (0.0)	2 (1.1)	0.691	0.165	0.0 (0.0)	1.8 (1.0)	0.34	0.223
Hepaticdysfunction (level 3-4, %)	1 (1.0)	4 (2.1)	0.381	0.227	0.4 (0.4)	3.9 (2.1)	0.384	0.237
Duration of surgery (minutes, mean (SD))	235.2 (61.6)	242.8 (48.46)	0.251	0.137	230.7 (63.1)	242.6 (46.9)	0.146	0.215
Blood loss (mL, mean (SD))	145.2 (98.3)	150.0 (99.8)	0.696	0.048	142.3 (99.9)	151.7 (91.3)	0.468	0.098
Pulmonary infection								
no	92 (91.1)	160 (85.6)	0.243	0.173	88.5 (92.3)	159.8 (86.9)	0.183	0.176
yes	9 (8.9)	27 (14.4)			7.4 (7.7)	24.0 (13.1)		
Pleural effusion								
no	79 (78.2)	150 (80.2)	0.804	0.049	77.6 (81.0)	141.9 (77.2)	0.524	0.091
yes	22 (21.8)	37 (19.8)			18.3 (19.0)	41.8 (22.8)		
Chylothorax								
no	100 (99.0)	183 (97.9)	0.811	0.093	95.2 (99.3)	179.9 (97.9)	0.305	0.119
yes	1 (1.0)	4 (2.1)			0.7 (0.7)	3.8 (2.1)		
Recurrent laryngeal nerve injury								
no	100 (99.0)	183 (97.9)	0.811	0.093	95.2 (99.3)	180.3 (98.1)	0.364	0.103
yes	1 (1.0)	4 (2.1)			0.7 (0.7)	3.4 (1.9)		
Anastomotic leak								
no	93 (92.1)	171 (91.4)	1	0.023	90.2 (94.0)	169.4 (92.2)	0.548	0.073
yes	8 (7.9)	16 (8.6)			5.7 (6.0)	14.4 (7.8)		
Postoperative hospital stay	11.1 (9.2)	12.7 (13.2)	0.3	0.135	10.9 (8.7)	12.5 (13.6)	0.256	0.139
Unplanned readmission								
no	99 (98.0)	185 (98.9)	0.918	0.074	94.8 (98.9)	182.1 (99.1)	0.796	0.026
yes	2 (2.0)	2 (1.1)			1.1 (1.1)	1.6 (0.9)		

Data are presented as n (%) or mean (SD); IPTW, Inverse probability of treatment weighting; SMD, Standardized mean differences.

### Pathology

Pathological outcomes are shown in **Table 3**. Before and after IPTW adjustment, the R0 resection rates were similar between the NICT and NCRT groups (97.0% vs. 96.3%, P = 0.996, SMD = 0.043; after IPTW: 96.9% vs. 97.0%, P = 0.97, SMD = 0.005). However, the NICT group had fewer patients with ypT0 (19.8% vs. 40.6%, P = 0.001, SMD = 0.563), ypN0 (59.4% vs. 71.1%, P = 0.039, SMD = 0.331), and yp stage I (41.6% vs. 59.9%, P = 0.012, SMD = 0.431). Before weighting, TRG and pCR also differed between groups; after weighting, these differences were attenuated, and pCR was no longer significantly different, whereas TRG showed only a marginal difference.

**Table 3:** Surgical and pathological findings.

	Before IPTW				After IPTW			
Variables	NICT	NCRT			NICT	NCRT		
	101	187	p	SMD	95.9	183.7	p	SMD
TRG (%)								
0	20 (19.8)	73 (39.0)	<0.001	0.575	26.0 (27.1)	72.1 (39.3)	0.051	0.376
1	24 (23.8)	48 (25.7)			26.7 (27.8)	45.4 (24.7)		
2	36 (35.6)	55 (29.4)			29.7 (31.0)	57.1 (31.1)		
3	21 (20.8)	11 (5.9)			13.5 (14.1)	9.1 (4.9)		
pCR (%)								
no	82 (81.2)	124 (66.3)	0.011	0.343	70.6 (73.6)	122.6 (66.7)	0.324	0.152
yes	19 (18.8)	63 (33.7)			25.3 (26.4)	61.2 (33.3)		
MPR (%)								
no	57 (56.4)	66 (35.3)	0.001	0.434	43.2 (45.1)	66.2 (36.0)	0.189	0.184
yes	44 (43.6)	121 (64.7)			52.7 (54.9)	117.5 (64.0)		
ypT (%)								
T0	20 (19.8)	76 (40.6)	0.001	0.563	26.0 (27.1)	74.1 (40.3)	0.105	0.393
T1	15 (14.9)	31 (16.6)			14.4 (15.0)	33.9 (18.5)		
T2	28 (27.7)	37 (19.8)			26.2 (27.3)	34.8 (18.9)		
T3	38 (37.6)	40 (21.4)			29.4 (30.6)	38.7 (21.1)		
T4	0 (0.0)	3 (1.6)			0.0 (0.0)	2.2 (1.2)		
ypN (%)								
N0	60 (59.4)	133 (71.1)	0.039	0.331	63.6 (66.3)	126.4 (68.8)	0.495	0.167
N1	23 (22.8)	38 (20.3)			18.8 (19.6)	36.1 (19.6)		
N2	16 (15.8)	16 (8.6)			12.3 (12.8)	21.3 (11.6)		
N3	2 (2.0)	0 (0.0)			1.2 (1.3)	0.0 (0.0)		
ypTNM (%)								
I	42 (41.6)	112 (59.9)	0.012	0.431	49.3 (51.4)	108.3 (58.9)	0.276	0.265
II	18 (17.8)	18 (9.6)			14.3 (14.9)	15.9 (8.6)		
IIIA	15 (14.9)	25 (13.4)			12.7 (13.2)	24.8 (13.5)		
IIIB	24 (23.8)	32 (17.1)			18.4 (19.2)	34.8 (19.0)		
IV	2 (2.0)	0 (0.0)			1.2 (1.3)	0.0 (0.0)		
Margin status (%)								
R0	98 (97.0)	180 (96.3)	0.996	0.043	92.9 (96.9)	178.2 (97.0)	0.97	0.005
R1/R2	3 (3.0)	7 (3.7)			3.0 (3.1)	5.5 (3.0)		

Data are presented as n (%); NICT, Neoadjuvant immuno-chemotherapy; NCRT, neoadjuvant chemoradiotherapy; pCR, Pathological Complete Response; MPR, Major Pathological response; TRG, Tumor regression grade; IPTW, Inverse probability of treatment weighting; SMD, Standardized mean differences.

### Survival

**Figure 4:**
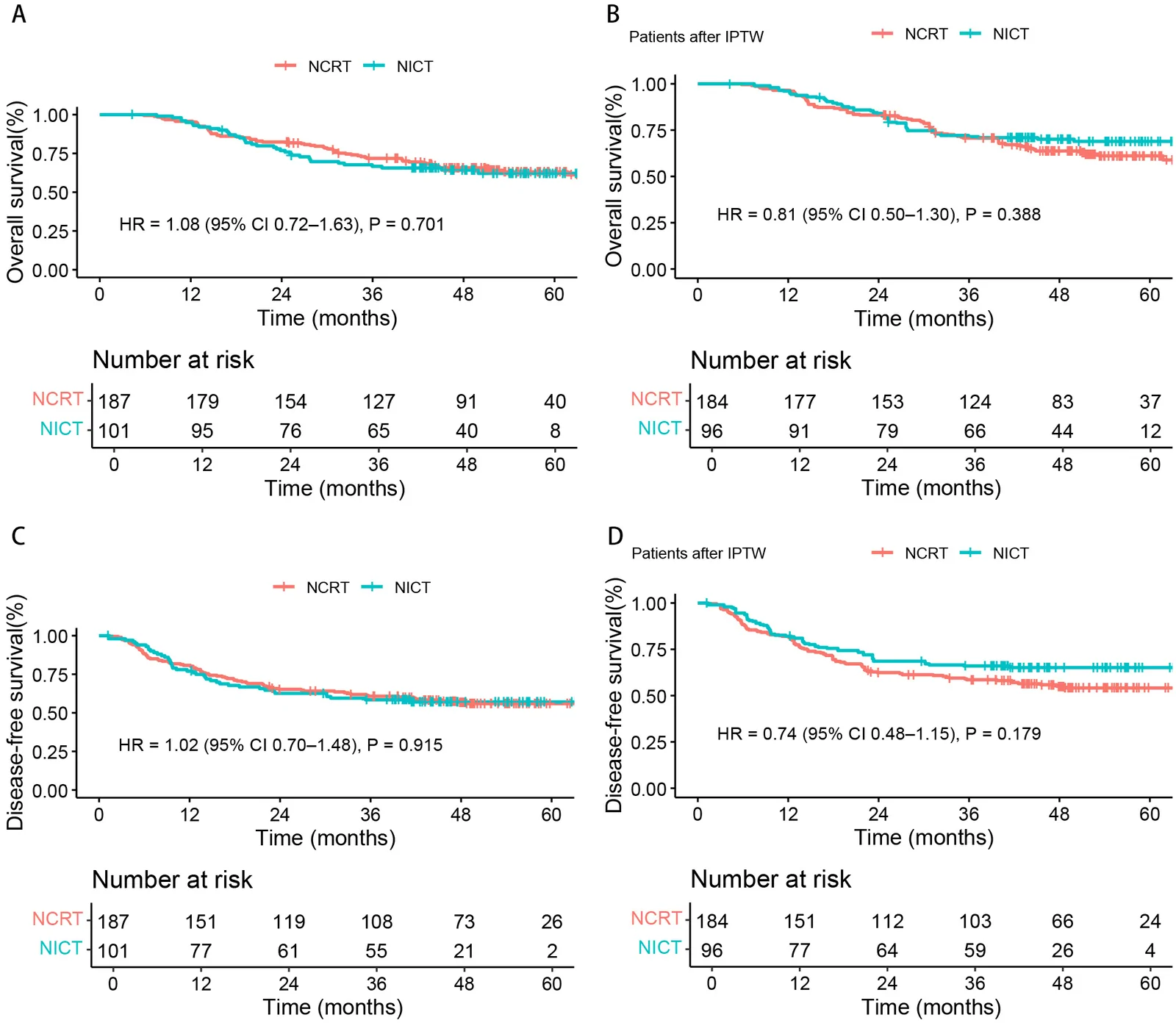
**(A)** Overall survival of NICT vs. NCRT group. **(B)** Overall survival of NICT vs. NCRT group after IPTW. **(C)** Disease-free survival of NICT vs. NCRT group. **(D)** Disease-free survival of NICT vs. NCRT group after IPTW. NICT, Neoadjuvant immunochemotherapy; NCRT, neoadjuvant chemoradiotherapy; HR, Hazard Ratio; CI, Confidence Interval; IPTW, Inverse probability of treatment weighting.

**Figure 5:**
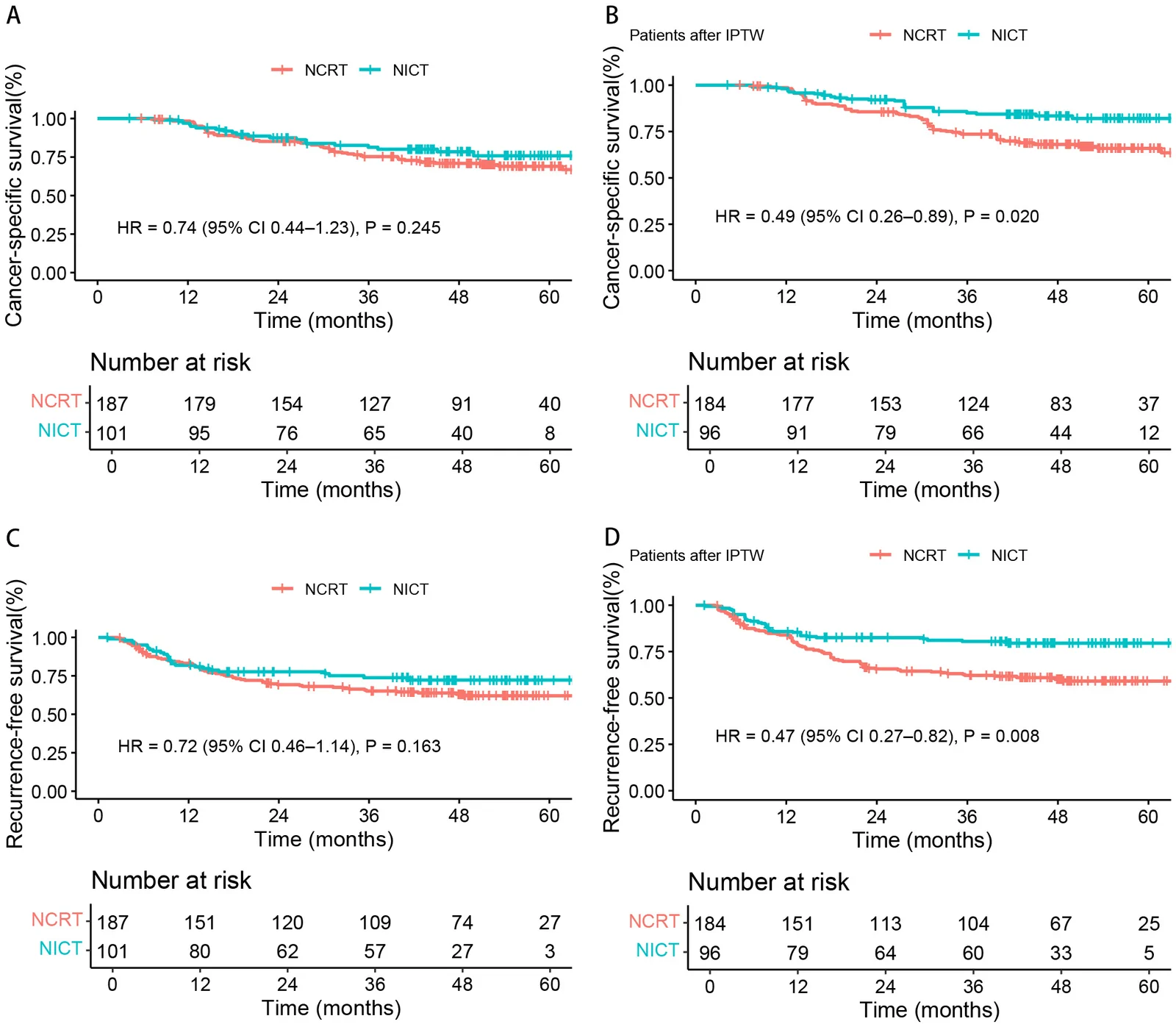
**(A)** Cancer-specific survival of NICT vs. NCRT group. **(B)** Cancer-specific survival of NICT vs. NCRT group after IPTW. **(C)** Recurrence-free survival of NICT vs. NCRT group. **(D)** Recurrence-free survival of NICT vs. NCRT group after IPTW. NICT, Neoadjuvant immunochemotherapy; NCRT, neoadjuvant chemoradiotherapy; HR, Hazard Ratio; CI, Confidence Interval; IPTW, Inverse probability of treatment weighting.

The median follow-up for the cohort was 49.3 months. Before and after IPTW adjustment, 5-year OS did not differ significantly between the NICT and NCRT groups (62.1% vs. 63.1%, HR = 1.08, 95% CI 0.72-1.63; P = 0.701, **Figure 4A**; after IPTW: 68.9% vs. 61.1%, HR = 0.81, 95% CI 0.50–1.30; P = 0.388, **Figure 4B**). Similarly, 5-year DFS was not significantly different between groups before or after IPTW (57.0% vs. 56.0%, HR = 1.02, 95% CI 0.70–1.48; P = 0.915, **Figure 4C**; after IPTW: 65.1% vs. 54.1%, HR = 0.74, 95% CI 0.48–1.15; P = 0.179, **Figure 4D**). However, as showing in **Figure 5**, NICT demonstrated a nonsignificant trend toward improved survival compared with NCRT (CSS: HR = 0.74, 95% CI 0.44-1.23; P = 0.245) and RFS: HR = 0.72, 95% CI 0.46-1.14; P = 0.163) before IPTW. Following the weighting, NICT was associated with significantly better outcomes. CSS improved with NICT (HR 0.49, 95% CI 0.26-0.89; P = 0.020), as did RFS (HR 0.47, 95% CI 0.27-0.82; P = 0.008).

In the pCR subgroup, the Kaplan–Meier curves for OS (HR = 0.22, 95% CI 0.03-1.68; P = 0.144; **Supplementary Figure 1A**), DFS (HR = 0.15, 95% CI 0.02–1.15; P = 0.068; **Supplementary Figure 1C**), CSS (HR = 0.26, 95% CI 0.03–1.97; P = 0.191; **Supplementary Figure 2A**) and RFS (HR = 0.17, 95% CI 0.02–1.30; P = 0.088; **Supplementary Figure 2C**) were completely separated, with the NICT group appearing to show a more favorable survival trend that did not reach statistical significance. After IPTW adjustment, the benefits in DFS (HR = 0.09, 95% CI 0.01-0.71; P = 0.023; **Supplementary Figure 1D**) and RFS (HR = 0.10, 95% CI 0.01-0.79; P = 0.029; **Supplementary Figure 2D**) reached statistical significance, while OS (HR = 0.13, 95% CI 0.02-1.09; P = 0.060; **Supplementary Figure 1B**) and CSS (HR = 0.15, 95% CI 0.02-1.28; P = 0.083; **Supplementary Figure 2B**) were borderline significant. At 60 months, compared with NCRT, the absolute survival rate improved by 18.5% to 25.9% (Effective bootstrap samples: 1864/2000), suggesting that NICT may confer a long-term outcome advantage. Although bootstrap analyses supported the stability of the effect direction and the absolute differences at time points, given the limited sample size and number of events, as well as multiplicity and model instability, the results should be considered hypothesis-generating only.

Among patients with non-pCR, unweighted Kaplan–Meier curves showed no significant differences in survival outcomes between NICT and NCRT (**Supplementary Figure 3A/C** or **Supplementary Figure 4A/C**). After IPTW, the curves separated in favor of NICT. The IPTW-adjusted HR for OS was 0.95 (95% CI 0.56–1.60; P = 0.839; **Supplementary Figure 3B**) and for DFS was 0.87 (95% CI 0.54–1.42; P = 0.583; **Supplementary Figure 3D**), indicating that neither endpoint reached statistical significance. In contrast, CSS showed a trend favoring NICT (HR = 0.55, 95% CI 0.28–1.05; P = 0.069; **Supplementary Figure 4B**), and RFS reached statistical significance (HR = 0.53, 95% CI 0.30–0.96; P = 0.035; **Supplementary Figure 4D**). Overall, among non-pCR patients, IPTW analysis suggests that compared with NCRT, NICT may reduce the risk of recurrence; however, the results are not stable and should be interpreted with caution.

### Cox regression analyses

In the multivariable models, NICT remained not significantly associated with OS or DFS but showed independent benefits for CSS (HR≈0.46, P = 0.011) and RFS (HR≈0.43, P = 0.002). Pathological nodal status (ypN) was the most consistent and strongest adverse prognostic factor across all outcomes (both N1 and N2/3 significantly increased risks for OS/DFS/CSS/RFS, most P values < 0.001). pCR was associated with better OS/DFS/CSS/RFS in univariable analysis but did not retain independent significance after multivariable adjustment. The specific details were presented in **Tables 4**–**7**.

**Table 4:** Univariate and multivariate COX regression analysis for OS.

Univariate and multivariate COX regression analysis								
	Univariate analysis				Multivariate analysis			
Variable	HR	CI.low	CI.high	p	HR	CI.low	CI.high	p
Group								
NCRT	Reference							
NICT	0.811	0.505	1.304	0.388				
Gender								
Male	Reference							
Female	0.785	0.474	1.299	0.345				
Age								
≤60	Reference							
>60	1.097	0.708	1.699	0.679				
Tumor location								
Upper	Reference							
Middle	1.135	0.543	2.369	0.737				
Lower	1.621	0.769	3.414	0.204				
Surgical approach								
McKeown	Reference							
Ivor Lewis	1.625	0.823	3.207	0.162				
RLNs	1.006	0.981	1.030	0.654				
TPS								
< 1%	Reference							
≥1%	1.281	0.690	2.379	0.432				
Unknown	1.281	0.730	2.249	0.388				
ypT								
T0	Reference				Reference			
T1	1.590	0.784	3.225	0.198	0.630	0.193	2.055	0.444
T2	1.775	0.940	3.350	0.077	0.704	0.217	2.283	0.559
T3-4	2.652	1.504	4.678	0.001	0.834	0.266	2.621	0.756
ypN								
N0	Reference				Reference			
N1	2.382	1.447	3.921	0.001	1.825	0.976	3.411	0.059
N2/3	4.099	2.529	6.641	<0.001	3.423	1.931	6.068	<0.001
Margin status								
R0	Reference							
R1/R2	1.689	0.758	3.765	0.200				
TRG								
0	Reference				Reference			
1	2.228	1.208	4.109	0.010	1.604	0.536	4.800	0.398
2	2.097	1.140	3.855	0.017	1.056	0.336	3.319	0.926
3	3.472	1.716	7.027	0.001	1.727	0.481	6.205	0.402
pCR								
No	Reference				Reference			
Yes	0.363	0.196	0.675	0.001	0.555	0.180	1.711	0.305

Data are presented as n (%); NICT, Neoadjuvant immuno-chemotherapy; NCRT, neoadjuvant chemoradiotherapy; HR, Hazard Ratio; CI, Confidence Interval; RLNs, retrieved lymph nodes; TPS, tumor proportion score; pCR, Pathological Complete Response; TRG, Tumor regression grade; OS, Overall Survival.

**Table 5:** Univariate and multivariate COX regression for DFS.

Univariate and multivariate COX regression analysis								
	Univariate analysis				Multivariate analysis			
Variable	HR	CI.low	CI.high	p	HR	CI.low	CI.high	p
Group								
NCRT	Reference							
NICT	0.740	0.477	1.148	0.179				
Gender								
Male	Reference							
Female	0.742	0.469	1.174	0.202				
Age								
≤60	Reference							
>60	0.997	0.667	1.490	0.988				
Tumor location								
Upper	Reference							
Middle	1.028	0.533	1.984	0.934				
Lower	1.465	0.751	2.858	0.262				
Surgical approach								
McKeown	Reference							
Ivor Lewis	1.303	0.673	2.523	0.433				
RLNs	1.008	0.986	1.031	0.470				
TPS								
< 1%	Reference							
≥1%	1.128	0.657	1.938	0.662				
Unknown	1.117	0.692	1.805	0.650				
ypT								
T0	Reference				Reference			
T1	1.721	0.919	3.221	0.090	0.926	0.311	2.754	0.890
T2	1.752	0.985	3.117	0.056	0.870	0.291	2.604	0.804
T3-4	2.556	1.520	4.296	<0.001	1.042	0.357	3.042	0.940
ypN								
N0	Reference				Reference			
N1	2.449	1.546	3.879	0.000	2.147	1.216	3.791	0.008
N2/3	4.297	2.637	7.001	<0.001	3.978	2.248	7.040	<0.001
Margin tatus								
R0	Reference							
R1/R2	1.662	0.802	3.442	0.172				
TRG								
0	Reference				Reference			
1	2.249	1.308	3.866	0.003	1.693	0.607	4.718	0.314
2	2.055	1.182	3.573	0.011	1.085	0.371	3.174	0.882
3	2.904	1.553	5.430	0.001	1.471	0.438	4.944	0.533
pCR								
No	Reference				Reference			
Yes	0.398	0.236	0.672	0.001	0.830	0.271	2.540	0.745

Data are presented as n (%); NICT, Neoadjuvant immuno-chemotherapy; NCRT, neoadjuvant chemoradiotherapy; HR, Hazard Ratio; CI, Confidence Interval; RLNs, retrieved lymph nodes; TPS, tumor proportion score; pCR, Pathological Complete Response; TRG, Tumor regression grade; DFS, Disease-Free Survival.

**Table 6:** Univariate and multivariate COX regression for CSS.

Univariate and multivariate COX regression analysis								
	Univariate analysis				Multivariate analysis			
Variable	HR	CI.low	CI.high	p	HR	CI.low	CI.high	p
Group								
NCRT	Reference				Reference			
NICT	0.485	0.263	0.894	0.020	0.460	0.253	0.839	0.011
Gender								
Male	Reference							
Female	0.891	0.498	1.593	0.697				
Age								
≤60	Reference							
>60	1.127	0.671	1.891	0.652				
Tumor location								
Upper	Reference							
Middle	1.096	0.472	2.547	0.831				
Lower	1.840	0.790	4.287	0.157				
Surgical approach								
McKeown	Reference							
Ivor Lewis	1.269	0.550	2.929	0.577				
RLNs	1.006	0.977	1.036	0.705				
TPS								
< 1%	Reference							
≥1%	1.444	0.687	3.035	0.332				
Unknown	1.322	0.666	2.623	0.425				
ypT								
T0	Reference				Reference			
T1	1.086	0.419	2.811	0.866	0.482	0.141	1.653	0.246
T2	1.343	0.641	2.814	0.435	0.620	0.193	1.984	0.420
T3-4	2.479	1.333	4.609	0.004	0.863	0.293	2.536	0.788
ypN								
N0	Reference				Reference			
N1	2.587	1.437	4.656	0.002	2.023	0.928	4.411	0.076
N2/3	5.355	3.049	9.403	<0.001	4.686	2.221	9.886	<0.001
Margin tatus								
R0	Reference							
R1/R2	1.509	0.536	4.242	0.436				
TRG								
0	Reference				Reference			
1	1.665	0.817	3.395	0.161	1.640	0.596	4.509	0.338
2	1.961	0.989	3.888	0.054	1.086	0.379	3.112	0.878
3	3.051	1.388	6.705	0.006	1.865	0.538	6.469	0.326
pCR								
No	Reference				Reference			
Yes	0.427	0.216	0.846	0.015	0.697	0.192	2.534	0.583

Data are presented as n (%); NICT, Neoadjuvant immuno-chemotherapy; NCRT, neoadjuvant chemoradiotherapy; HR, Hazard Ratio; CI, Confidence Interval; RLNs, retrieved lymph nodes; TPS, tumor proportion score; pCR, Pathological Complete Response; TRG, Tumor regression grade; CSS, Cancer-Specific Survival.

**Table 7:** Univariate and multivariate COX regression for RFS.

Univariate and multivariate COX regression analysis								
	Univariate analysis				Multivariate analysis			
Variable	HR	CI.low	CI.high	p	HR	CI.low	CI.high	p
Group								
NCRT	Reference				Reference			
NICT	0.474	0.274	0.820	0.008	0.430	0.250	0.741	0.002
Gender								
Male	Reference							
Female	0.818	0.486	1.375	0.448				
Age								
≤60	Reference							
>60	0.998	0.630	1.579	0.992				
Tumor location								
Upper	Reference							
Middle	0.954	0.459	1.980	0.899				
Lower	1.521	0.732	3.161	0.261				
Surgical approach								
McKeown	Reference							
Ivor Lewis	0.957	0.419	2.187	0.917				
RLNs	1.008	0.984	1.032	0.515				
TPS								
< 1%	Reference							
≥1%	1.174	0.637	2.163	0.607				
Unknown	1.059	0.608	1.843	0.840				
ypT								
T0	Reference				Reference			
T1	1.205	0.554	2.621	0.638	0.775	0.253	2.370	0.654
T2	1.348	0.697	2.605	0.375	0.845	0.275	2.595	0.769
T3-4	2.281	1.317	3.950	0.003	1.183	0.431	3.243	0.744
ypN								
N0	Reference				Reference			
N1	2.474	1.454	4.210	0.001	2.356	1.169	4.751	0.017
N2/3	4.822	2.851	8.155	<0.001	4.726	2.401	9.304	<0.001
Margin tatus								
R0	Reference							
R1/R2	1.512	0.616	3.712	0.367				
TRG								
0	Reference				Reference			
1	1.683	0.910	3.116	0.097	1.730	0.662	4.520	0.264
2	1.849	1.015	3.366	0.044	1.131	0.411	3.118	0.811
3	2.400	1.204	4.786	0.013	1.583	0.485	5.168	0.447
pCR								
No	Reference				Reference			
Yes	0.488	0.279	0.852	0.012	1.117	0.326	3.832	0.860

Data are presented as n (%); NICT, Neoadjuvant immuno-chemotherapy; NCRT, neoadjuvant chemoradiotherapy; HR, Hazard Ratio; CI, Confidence Interval; RLNs, retrieved lymph nodes; TPS, tumor proportion score; pCR, Pathological Complete Response; TRG, Tumor regression grade; RFS, Recurrence-Free Survival.

### Recurrence profile

Integrating **Table 8** and **Supplementary Figure 5**, NICT was associated with a lower recurrence burden overall, driven mainly by fewer distant metastases. Local recurrence remained low in both groups and did not differ significantly. After weighting, overall recurrence and distant metastasis were both less frequent in the NICT group, whereas local control remained comparable between groups. Considering the sample and event sizes and the dependence on the model, these findings are primarily hypothesis-generating and warrant further validation.

**Table 8:** Incidence of recurrence.

	Before IPTW				After IPTW			
Variables	NICT (n=101)	NCRT (n=187)	p	SMD	NICT (n=95.9)	NCRT (n=183.7)	p	SMD
Overall recurrence	26 (25.7)	67 (35.9)	0.209	0.223			0.004	0.443
Local recurrence	7 (6.9)	16 (8.6)			4.1 (4.3)	15.5 (8.4)		
Distant recurrence	19 (18.8)	51 (27.3)			14.3 (14.9)	55.9 (30.4)		

## Discussion

This target-trial emulation compared two prespecified neoadjuvant strategies for locally advanced thoracic ESCC: NICT and NCRT. In the weighted analysis, NICT did not confer a statistically significant improvement in OS or DFS compared with NCRT. These findings suggest that, when analyzed as a head-to-head treatment strategy comparison under a target-trial framework, NICT provides survival outcomes that are at least comparable to those of conventional NCRT in this population.

### Comparison with previous studies

A key finding was the difference in toxicity and pathological response profiles between the two strategies. Compared with NICT, NCRT was associated with significantly higher rates of grade 3–4 myelosuppression (leukopenia, neutropenia, and lymphocytopenia), and this difference remained robust after IPTW. This finding is consistent with prior studies (11, 12). Although this hematologic toxicity was more frequent, it did not translate into a higher rate of perioperative complications or greater resource utilization, suggesting that the excess toxicity was clinically manageable in the perioperative setting.

The pathological response pattern also differed between strategies. Reported pCR rates after NICT range roughly from 20.2% to 39.2% (15, 18–20); in our study, the pCR rate was lower in the NICT group than in the NCRT group (18.8% vs 33.7%, p=0.011). NCRT produced higher pCR and ypT0/TRG 0 rates, which is biologically plausible given the direct cytotoxic effect of radiotherapy on the primary tumor and regional lymph nodes (21, 22). Notably, in our cohort and in trials such as CROSS and NEOCRTEC-5010 (4, 5), pCR after NCRT did not preclude distant metastasis, the most common failure pattern; CMISG1701 likewise showed that superior tumor response does not necessarily translate into survival benefit (23). By contrast, NICT yielded a lower pathological response rate but appeared to preserve long-term disease control. This apparent disconnect between short-term pathologic response and long-term outcome is increasingly recognized in the immunotherapy era. NCRT may achieve deeper local pathological remission primarily through the direct cytotoxic effects of radiotherapy on the primary tumor and regional lymph nodes. However, pathological response at the time of surgery mainly reflects residual local disease and does not necessarily indicate whether micrometastatic deposits have been eradicated. In ESCC, both complete locoregional resection and effective systemic disease control are critical therapeutic objectives. With the widespread adoption of neoadjuvant strategies and more extensive lymphadenectomy, a substantial proportion of patients treated with either NCRT or NICT can achieve complete clearance of regional disease; nonetheless, distant metastasis remains the predominant pattern of postoperative failure. Therefore, long-term recurrence and cancer-related mortality are determined to a greater extent by control of systemic micrometastatic disease. NICT may provide more durable systemic antitumor activity through immune activation, the formation of immune memory, and ongoing immune surveillance against micrometastatic foci. In the era of immunotherapy, pCR may be a less reliable surrogate endpoint for long-term survival, because the benefit of immunotherapy may not manifest as a higher rate of immediate pathological tumor eradication, but rather as slower yet more sustained suppression of recurrence.

Our findings are broadly consistent with previous reports showing that neoadjuvant immunotherapy is feasible and produces meaningful antitumor activity in locally advanced ESCC. Some recent studies have suggested that NICT may offer superior long-term outcomes compared with NCRT (13, 19); meanwhile, several smaller studies suggest that NICT may provide long-term survival outcomes only comparable to those of NCRT (14, 15, 24). Similarly, we did not observe an OS advantage for NICT in the full cohort. However, we observed a significant advantage of NICT in tumor-related outcomes (CSS and RFS) after IPTW. The findings are consistent with the multicenter study by Guo et al. (13), which reported that ICIs significantly reduce the risk of recurrence. Guo et al. had a larger sample size and more events, enabling greater sensitivity to detect differences. Similarly, Zhu et al. reported that, after propensity score matching, the NICT group demonstrated a trend toward better distant metastasis-free survival than the NCRT group, although the difference did not reach statistical significance (24). These findings may further support the potential systemic benefit of NICT. In this context, our study adds value by explicitly emulating a target trial, using stabilized IPTW, weight trimming, and balance diagnostics to reduce confounding from nonrandom treatment allocation. We also reported ESS and performed bootstrap-based sensitivity analyses in small subgroups to present uncertainty more transparently.

The exploratory analyses deserve cautious interpretation. In the exploratory subgroup analysis, Kaplan–Meier curves appeared to separate and bootstrap-weighted Cox estimates favored NICT—the bootstrap analyses also suggested a potential advantage of NICT over NCRT for DFS and RFS (Cox HR ≈ 0.09-0.10; 23.7-25.9% absolute gain at 60 months), while OS and CSS showed borderline trends. These findings are hypothesis-generating and may suggest that even among apparent complete responders, the systemic immune effects of NICT may contribute to more durable disease control. However, the small number of events and limited subgroup size mean that these results should not be overinterpreted. They require validation in larger cohorts with prospective design and longer follow-up.

### Clinical implications

From a clinical perspective, the present results suggest that NICT may be a reasonable alternative to NCRT for eligible patients with locally advanced thoracic ESCC, particularly when reducing hematologic toxicity is an important consideration. NCRT may still offer an advantage in locoregional tumor sterilization and pCR achievement, whereas NICT may better support durable systemic control. Therefore, treatment selection should balance the need for local control against toxicity tolerance and the risk of distant relapse. For patients with high-risk local disease, treatment intensification focused on local control may still be warranted; for those with greater systemic relapse risk, immunotherapy-based strategies may be more attractive.

### Future directions

A major aim for future research is to better identify the subset of patients who will truly benefit, thereby maximizing survival gains. Serial circulating tumor DNA assessment for minimal residual disease, immune profiling of tumor and peripheral blood, and spatial analyses of the tumor microenvironment are needed to clarify mechanisms, identify beneficiaries, and evaluate comparative strategies including NICT, NCRT, and combined ICI-radiotherapy approaches. In parallel, the gradual integration of immunotherapy with radiotherapy in ESCC is gaining support from both clinical and preclinical evidence. Radiotherapy provides strong locoregional control and may promote antitumor immunity by enhancing antigen release, antigen presentation, and T-cell infiltration, while preclinical studies suggest that ionizing radiation can act synergistically with ICIs to overcome immune resistance and reshape the tumor microenvironment (11, 25–29). Accordingly, adding radiotherapy to an NICT backbone merits investigation as a strategy to improve pathological response and systemic disease control.

Preliminary results from a multicenter trial from our institution, presented at American Society of Clinical Oncology (ASCO) Gastrointestinal Cancers Symposium 2025, showed that adding radiotherapy to NICT produced a pCR rate of approximately 60% with an acceptable safety profile (DOI: 10.1200/JCO.2025.43.4_suppl.LBA329); the long-term outcomes of these patients are awaited. Such results support the emerging view that induction chemoimmunotherapy may serve as a practical platform for subsequent radiotherapy integration, potentially enabling deeper tumor regression and more durable disease control. Concurrent translational studies, including circulating tumor DNA assays for minimal residual disease, will be valuable for identifying patients most likely to benefit from immunotherapy-based neoadjuvant strategies and for defining when radiotherapy is necessary to optimize local control.

### Limitations

Several limitations should be acknowledged. First, as an observational study, residual confounding cannot be excluded, even under a target-trial framework and after IPTW adjustment. Second, treatment allocation was influenced by calendar time and clinical judgment, which may have introduced channeling bias. Third, important biological factors such as PD-L1 expression, circulating tumor DNA, and immune microenvironment features were not uniformly available, limiting mechanistic interpretation and the ability to identify effect modifiers. Future prospective studies with biomarker integration are needed to validate these findings and clarify which patients derive the greatest benefit from each neoadjuvant strategy.

## Conclusion

NICT and NCRT showed broadly comparable long-term OS and DFS, but their pathological response patterns differed. NCRT was associated with deeper local pathological remission, whereas NICT had a more favorable safety profile and significantly lower rates of severe hematologic toxicity. After IPTW adjustment, NICT was associated with improved cancer-specific survival and recurrence-free survival, primarily through reducing overall recurrence—especially distant metastasis.

## Data Availability

The raw data supporting the conclusions of this article will be made available by the authors, without undue reservation.

## Supplementary material

Supplementary Figure 1 **(A)** Overall survival of NICT vs. NCRT group for pCR patients. **(B)** Overall survival of NICT vs. NCRT group for pCR patients after IPTW. **(C)** Disease-free survival of NICT vs. NCRT group for pCR patients. **(D)** Disease-free survival of NICT vs. NCRT group for pCR patients after IPTW. NICT, Neoadjuvant immunochemotherapy; NCRT, neoadjuvant chemoradiotherapy; HR, Hazard Ratio; CI, Confidence Interval; IPTW, Inverse probability of treatment weighting; pCR, Pathological Complete Response.

Supplementary Figure 2 **(A)** Cancer-specific survival of NICT vs. NCRT group for pCR patients. **(B)** Cancer-specific survival of NICT vs. NCRT group for pCR patients after IPTW. **(C)** Recurrence-free survival of NICT vs. NCRT group for pCR patients. **(D)** Recurrence-free survival of NICT vs. NCRT group for pCR patients after IPTW. NICT, Neoadjuvant immunochemotherapy; NCRT, neoadjuvant chemoradiotherapy; HR, Hazard Ratio; CI, Confidence Interval; IPTW, Inverse probability of treatment weighting; pCR, Pathological Complete Response.

Supplementary Figure 3 **(A)** Overall survival of NICT vs. NCRT group for non-pCR patients. **(B)** Overall survival of NICT vs. NCRT group for non-pCR patients after IPTW. **(C)** Disease-free survival of NICT vs. NCRT group for non-pCR patients. **(D)** Disease-free survival of NICT vs. NCRT group for non-pCR patients after IPTW. NICT, Neoadjuvant immunochemotherapy; NCRT, neoadjuvant chemoradiotherapy; HR, Hazard Ratio; CI, Confidence Interval; IPTW, Inverse probability of treatment weighting; pCR, Pathological Complete Response.

Supplementary Figure 4 **(A)** Cancer-specific survival of NICT vs. NCRT group for non-pCR patients. **(B)** Cancer-specific survival of NICT vs. NCRT group for non-pCR patients after IPTW. **(C)** Recurrence-free survival of NICT vs. NCRT group for non-pCR patients. **(D)** Recurrence-free survival of NICT vs. NCRT group for non-pCR patients after IPTW. NICT, Neoadjuvant immunochemotherapy; NCRT, neoadjuvant chemoradiotherapy; HR, Hazard Ratio; CI, Confidence Interval; IPTW, Inverse probability of treatment weighting; pCR, Pathological Complete Response.

Supplementary Figure 5 **(A)** Local recurrence-free survival of NICT vs. NCRT group. **(B)** Local recurrence-free survival NICT vs. NCRT group after IPTW. **(C)** Distant metastasis-free survival of NICT vs. NCRT group. **(D)** Distant metastasis-free survival of NICT vs. NCRT group after IPTW. NICT, Neoadjuvant immunochemotherapy; NCRT, neoadjuvant chemoradiotherapy; HR, Hazard Ratio; CI, Confidence Interval; IPTW, Inverse probability of treatment weighting.

Supplementary Figure 6 IPTW-weighted log-minus-log survival curves for overall and disease-related survival outcomes.

Supplementary Figure 7 Schoenfeld residual-based assessment of the proportional hazards assumption after IPTW.
